# Clinical pharmacokinetic and in vitro combination studies of nolatrexed dihydrochloride (AG337, Thymitaq^TM^) and paclitaxel

**DOI:** 10.1054/bjoc.2000.1172

**Published:** 2000-04-03

**Authors:** A N Hughes, M J Griffin, D R Newell, A H Calvert, A Johnston, B Kerr, C Lee, B Liang, A V Boddy

**Affiliations:** Cancer Research Unit, Medical School, University of Newcastle upon Tyne, Newcastle upon Tyne, NE2 4HH, UK; Department of Medical Oncology, Newcastle General Hospital, Westgate Road, Newcastle upon Tyne, NE4 6BE, UK; Agouron Pharmaceuticals, La Jolla, CA, USA

**Keywords:** metabolism, interaction, schedule, inhibition

## Abstract

A clinical study of nolatrexed dihydrochloride (AG337, Thymitaq^TM^) in combination with paclitaxel was performed. The aims were to optimize the schedule of administration and determine any pharmacokinetic (PK) interactions between the two drugs. In vitro combination studies were performed to assist with schedule optimization. Three patients were entered on each of three different schedules of administration of the two drugs: (1) paclitaxel 0–3 h, nolatrexed 24–144 h; (2) nolatrexed 0–120 h, paclitaxel 48–51 h; (3) nolatrexed 0–120 h, paclitaxel 126–129 h. Paclitaxel was administered at a dose of 80 mg m^−2^over 3 h and nolatrexed at a dose of 500 mg m^−2^day^−1^as a 120-h continuous intravenous infusion. Plasma concentrations of both drugs were determined by high performance liquid chromatography. In vitro growth inhibition studies using corresponding schedules were performed using two head and neck cancer cell lines. In both HNX14C and HNX22B cell lines, synergistic growth inhibition was observed on schedule 2, whereas schedules 1 and 3 demonstrated antagonistic effects. In the clinical study, there was no effect of schedule on the pharmacokinetics of nolatrexed. However, patients on schedules 1 and 3 had a higher clearance of paclitaxel (322–520 ml min^−1^m^−2^) than those on schedule 2 (165–238 ml min^−1^m^−2^). Peak plasma concentrations (1.66–1.93 vs 0.86–1.32 μM) and areas under the curve (392–565 vs 180–291 μM min^−1^) of paclitaxel were correspondingly higher on schedule 2. The pharmacokinetic interaction was confirmed by studies with human liver microsomes, nolatrexed being an inhibitor of the major routes of metabolism of paclitaxel. Toxicity was not schedule-dependent. Nolatrexed and paclitaxel may be safely given together when administered sequentially at the doses used in this study. Studies in vitro suggest some synergy, however, due to a pharmacokinetic interaction, paclitaxel doses should be reduced when administered during nolatrexed infusion. © 2000 Cancer Research Campaign


British Journal of Cancer (2000) 82(9), 1519–1527
© 2000 Cancer Research Campaign
DOI: 10.1054/ bjoc.2000.1172, available online at http://www.idealibrary.com on IDEAL®

### Clinical pharmacokinetic and in vitro combination studies of nolatrexed dihydrochloride (AG337, Thymitaq™) and paclitaxel

**AN Hughes[1,2], MJ Griffin[1], DR Newell[1], AH Calvert[1,2], A Johnston[3], B Kerr[3], C Lee[3], B Liang[3] and AV Boddy[1]**

[1]Cancer Research Unit, Medical School, University of Newcastle upon Tyne, Newcastle upon Tyne NE2 4HH, UK; [2]Department of Medical Oncology, Newcastle General Hospital, Westgate Road, Newcastle upon Tyne NE4 6BE, UK; [3]Agouron Pharmaceuticals, La Jolla, CA, USA

**Summary** A clinical study of nolatrexed dihydrochloride (AG337, Thymitaq™) in combination with paclitaxel was performed. The aims were to optimize the schedule of administration and determine any pharmacokinetic (PK) interactions between the two drugs. In vitro combination studies were performed to assist with schedule optimization. Three patients were entered on each of three different schedules of administration of the two drugs: (1) paclitaxel 0–3 h, nolatrexed 24–144 h; (2) nolatrexed 0–120 h, paclitaxel 48–51 h; (3) nolatrexed 0–120 h, paclitaxel 126–129 h. Paclitaxel was administered at a dose of 80 mg m$^{-2}$ over 3 h and nolatrexed at a dose of 500 mg m$^{-2}$ day$^{-1}$ as a 120-h continuous intravenous infusion. Plasma concentrations of both drugs were determined by high performance liquid chromatography. In vitro growth inhibition studies using corresponding schedules were performed using two head and neck cancer cell lines. In both HNX14C and HNX22B cell lines, synergistic growth inhibition was observed on schedule 2, whereas schedules 1 and 3 demonstrated antagonistic effects. In the clinical study, there was no effect of schedule on the pharmacokinetics of nolatrexed. However, patients on schedules 1 and 3 had a higher clearance of paclitaxel (322–520 ml min$^{-1}$ m$^{-2}$) than those on schedule 2 (165–238 ml min$^{-1}$ m$^{-2}$). Peak plasma concentrations (1.66–1.93 vs 0.86–1.32 µM) and areas under the curve (392–565 vs 180–291 µM min$^{-1}$) of paclitaxel were correspondingly higher on schedule 2. The pharmacokinetic interaction was confirmed by studies with human liver microsomes, nolatrexed being an inhibitor of the major routes of metabolism of paclitaxel. Toxicity was not schedule-dependent. Nolatrexed and paclitaxel may be safely given together when administered sequentially at the doses used in this study. Studies in vitro suggest some synergy, however, due to a pharmacokinetic interaction, paclitaxel doses should be reduced when administered during nolatrexed infusion. © 2000 Cancer Research Campaign

**Keywords**: metabolism; interaction; schedule; inhibition

Nolatrexed dihydrochloride (AG337, Thymitaq™) was designed by Agouron Pharmaceuticals, La Jolla, CA, USA using knowledge of the three-dimensional structure of the active site of thymidylate synthase (TS) (Webber et al, 1993). It acts at the folate-binding site of the TS enzyme and, as a non-classical antifolate, the activity of nolatrexed is not dependent on activation by the enzyme folyl polyglutamate synthetase (FPGS) or a specific transport mechanism for cellular uptake. Previous studies with nolatrexed have shown that the optimal schedule of administration is as a continuous intravenous infusion over 5 days at a dose of 800 mg m$^{-2}$ day$^{-1}$ (Rafi et al, 1995, 1998). Clinical activity has been demonstrated in head and neck cancer (Belani et al, 1997*a*), hepatoma (Stuart et al, 1996), pancreatic carcinoma (Loh et al, 1997) and colonic carcinoma (Belani et al, 1997*b*). The main toxicities associated with nolatrexed are haematological and gastrointestinal (Rafi et al, 1998).

Paclitaxel, derived from the Pacific yew tree *Taxus brevifolia*, acts by stabilizing microtubules against depolymerization. Paclitaxel has demonstrated anti-tumour activity against a wide variety of different tumour types, most impressively ovarian (Goldspiel, 1997) and breast carcinoma (D'Andrea and Seidman, 1997; Goldspiel, 1997; Wilson et al, 1994). However, it has also shown significant activity against squamous cell carcinoma of the head and neck (Aisner and Cortes-Funes, 1997; Vokes et al, 1995). Major paclitaxel side-effects include myelosuppression, total alopecia and paraesthesiae (Rowinsky, 1997).

In view of the activity of both nolatrexed and paclitaxel in head and neck cancer and, with the exception of myelosuppression, their non-overlapping toxicities, the combination of the two drugs has been explored in clinical and laboratory-based studies. As the schedule of administration of a combination of drugs can play a major role in the toxicity and activity, three different schedules of drug administration were explored. On schedule 1, paclitaxel was administered prior to nolatrexed and on schedule 3 the order was reversed. On schedule 2, the paclitaxel was administered in the middle of the nolatrexed infusion. The pharmacokinetic results obtained for the patients on schedule 2 were of particular interest given that both paclitaxel and nolatrexed undergo metabolism mediated by the cytochrome P450 system.

The aims of the clinical study were:

1. To determine whether nolatrexed and paclitaxel could be safely given in combination
2. To assess the toxicity of the combination and to record any anti-tumour activity
3. To optimize the schedule of administration of the two agents

*Received 30 July 1999*
*Revised 13 January 2000*
*Accepted 18 January 2000*

*Correspondence to*: AN Hughes

**Table 1** Patient characteristics and outcome

| Patient no. | Age | Sex | Diagnosis | Schedule | Prior chemo regimes | No. of courses | Response |
|---|---|---|---|---|---|---|---|
| 3 | 52 | F | Adrenocortical | 1 | 3 | 6 | SD |
| 6 | 47 | F | Colorectal | 1 | 2 | 1 | PD |
| 9 | 65 | F | Melanoma | 1 | None | 4 | SD |
| 5 | 45 | F | NSCLC | 2 | 1 | 4 | SD |
| 7 | 40 | F | *Adenocarcinoma | 2 | None | 2 | PD |
| 10 | 72 | M | Caecal | 2 | 1 | 1 | NE |
| 2 | 59 | F | Ovarian | 3 | 2 | 1 | NE |
| 4 | 56 | F | Ovarian | 3 | 3 | 2 | PD |
| 8 | 44 | F | Breast | 3 | 1 | 1 | NE |

Patient 1 never commenced treatment due to deteriorating clinical condition. NSCLC = non-small-cell lung cancer. * = unknown primary site. SD = stable disease, PD = progressive disease, NE = not evaluable for response.

4. To determine if there was any pharmacokinetic interaction between the two drugs.

In vitro studies were performed using two head and neck cancer cell lines and human liver microsomes to investigate the growth inhibitory effects and possible interactions for the same schedules of drug administration as used in the clinical study.

### MATERIALS AND METHODS

### Materials

Nolatrexed and associated materials were obtained as described previously (Rafi et al, 1995). Paclitaxel was kindly supplied by Bristol Myers Squibb (Princeton, NJ, USA).

For the tissue culture work, RPMI-1640 medium complete and trypsin were supplied by Gibco-BRL, Life Technologies (Paisley, UK) and fetal calf serum by Globe Pharmaceuticals (Surrey, UK). EDTA, trichloroacetic acid, TRIS base, sulphorhodamine B and dimethyl sulphoxide (DMSO) were all obtained from Sigma Chemical Company (Poole, Dorset, UK). Glacial acetic acid and dialysis membrane was supplied by BDH (Dorset, UK). Life Technologies (Paisley, UK) also supplied the NUNC 96-well plates which were read on a Dynatech MR7000 microtitre plate reader (Dynatech, Guernsey, UK). The two head and neck cancer cell lines, HNX14C and HNX22B, were kindly provided by Dr Braakhuis (Free University, Amsterdam, The Netherlands).

### In vitro combination studies

The two head and neck cell lines, HNX14C and HNX22B, were cultured at 37°C in a humidified atmosphere of 95% air/5% carbon dioxide in RPMI-1640 medium containing 10% v/v dialysed fetal calf serum, supplemented with glutamine and sodium bicarbonate. Both cell lines were passaged twice weekly using trypsin–EDTA. Initial studies were performed to determine the $IC_{50}$ for nolatrexed (120-h exposure) and paclitaxel (3-h exposure) for each cell line. For each experiment, exponentially growing cells were washed, trypsinized and seeded at a concentration of $5 \times 10^3$ ml$^{-1}$ (HNX14C) or $1 \times 10^4$ ml$^{-1}$ (HNX22B) into the wells of a 96-well plate. Cells were then left to adhere for 24 h before drug treatment. For each cell line, 3 different schedules of drug combination, corresponding to those used in the clinical study, were studied.

#### *Schedule 1: paclitaxel 0–3 h, nolatrexed 24–144 h*

After the initial 24 h, cells were exposed to varying concentrations of paclitaxel for 3 h. The drug was removed and the plates were then left for 21 h, after which varying concentrations of nolatrexed were added. The paclitaxel and nolatrexed drug concentrations used were as follows: $0.01 \times IC_{50}$ for the drug as a single agent, $0.1 \times IC_{50}$, $0.3 \times IC_{50}$, $IC_{50}$, $3 \times IC_{50}$, $10 \times IC_{50}$. The ratio of drug concentrations at each dose was therefore the same. Cell growth inhibition was then assessed at 6 days (144 h) by the sulphorhodamine B (SRB) assay (Skehan et al, 1990). For each cell line, experiments were repeated on a minimum of three occasions.

#### *Schedule 2: nolatrexed 0–120 h, paclitaxel 48–51 h*

Cells were seeded as above and identical nolatrexed concentrations as those used in schedule 1 were added 24 h after cell adherence. At 48 h, the nolatrexed was removed and replaced by a combination of both nolatrexed and paclitaxel. This was replaced with nolatrexed alone 3 h later. Duplicate plates were used for this schedule and SRB assays were performed at day 6 (144 h) and day 8 (192 h). Experiments were repeated at least three times.

#### *Schedule 3: nolatrexed 0–120 h, paclitaxel 126–129 h*

Triplicate plates were set up and cells were exposed to nolatrexed for 120 h. The nolatrexed was then removed and replaced by paclitaxel 6 h later. At 129 h, the paclitaxel was removed. SRB assays were performed on days 6, 8 and 10 (144, 192 and 240 h). Experiments were repeated at least three times.

On every 96-well plate for each schedule, control columns of cells were exposed to medium alone, medium/DMSO (the paclitaxel was diluted in DMSO) and single-agent drug $IC_{50}$ concentrations.

The effects of the drug combinations were evaluated using the method of Chou and Talalay (1984). For each schedule of drug administration for both cell lines, curves were plotted of the log of the combination index (CI) versus the fraction of cells affected (Fa).

### Patient population

In total, nine patients were available for study, three on each of the 3 schedules of drug administration. Individual patient characteristics are shown in Table 1. Eight of the patients were female, age ranged from 40 to 72 years, and a number of different tumour

 © 2000 Cancer Research Campaign types were treated. Only two of the nine patients were chemotherapy naive. Twenty-two courses of treatment were administered in total. The study was approved by the Regional Ethical Committee. All patients gave written informed consent prior to registration on the study. Study exclusion and inclusion criteria were as described previously (Rafi et al, 1995), with the additional exclusion of patients having previously been treated with paclitaxel or nolatrexed. Although ultimately intended for use in patients with head and neck cancer, this initial study was performed in any patient with a solid tumour that was not amenable to conventional therapy.

### Patient monitoring

Before treatment, a complete history was taken and a physical examination with evaluation of WHO performance status performed. A full blood count, urea and electrolyte concentrations and liver function tests were measured and, in addition, an electrocardiogram (ECG) and EDTA clearance determination were obtained. All but the latter two tests were repeated twice weekly during the first course of treatment and weekly thereafter. Although the primary purpose of the study was not to assess tumour response, patients with measurable or evaluable disease had representative lesions assessed at least every two courses by physical examination or appropriate radiological studies. Treatment courses were repeated every 3 or 4 weeks depending on recovery of the patient from the previous course. Treatment was continued to a maximum six courses. Patients were withdrawn from the study in the event of progressive disease, or if it was felt by the investigator to be in the best interest of the patient. Common toxicity criteria (CTC) for toxicity and response were used.

### Clinical and drug metabolism studies

Nine patients were randomized to one of three different schedules of administration of nolatrexed and paclitaxel:

Schedule 1: paclitaxel 0–3 h, nolatrexed 24–144 h
Schedule 2: nolatrexed 0–120 h, paclitaxel 48–51 h
Schedule 3: nolatrexed 0–120 h, paclitaxel 126–129 h.

Three patients were treated on each schedule.

Nolatrexed was administered as a continuous 120-h intravenous infusion via a central venous catheter at a dose of 500 mg $m^{-2}$ $day^{-1}$. The total dose was diluted in normal saline and slowly administered over the 5-day period using a Baxter pump (Baxter Healthcare, Newbury, Berks, UK). Paclitaxel was prepared according to the instructions of the manufacturer and administered as a 3-h intravenous infusion in 5% w/v dextrose at a dose of 80 mg $m^{-2}$. In order to prevent hypersensitivity reactions to the paclitaxel, patients were pre-medicated with dexamethasone, chlorpheniramine and cimetidine. Courses of treatment were repeated every 3–4 weeks depending on the recovery of the patient from the prior course.

Pharmacokinetic sampling was performed during the first course of treatment in all nine patients. The timing of samples was designed such that data from different schedules were comparable. Plasma samples were stored at –20°C until analysis by methods described previously (Rafi et al, 1995; Siddiqui et al, 1997).

In vitro studies investigating the effect of nolatrexed on the metabolism of paclitaxel were conducted using human liver microsomes (Keystone Skin Bank Exton, PA, USA). Ten individual human liver microsome preparations with P450 content (determined by CO difference spectra) greater than 0.3 nmol $mg^{-1}$ were pooled together based on equivalent milligrams of protein (determined by the Pierce Kit, Pierce Rockford, IL, USA). This pooling method ensured that there was equal contribution of the cytochrome P450 isoforms from each liver since the protein concentrations from each sample varied from 11.6 to 28.3 mg $ml^{-1}$.

Pooled human liver microsomes (1 mg protein per ml) were incubated with paclitaxel (for preliminary study 40, 80 and 200 μM and for $K_i$ determination 2.5, 5, 20 μM) and nolatrexed (for preliminary study 0–10 μM and for $K_i$ determination 0–2.0 μM) in 100 mM potassium phosphate buffer, pH 7.4, at 37°C in a shaking water bath for 5 min before initiating the reaction with NADPH (2 mM final concentration). A control sample containing all reagents except nolatrexed was also included. The final volume of each incubation was 0.5 ml. The reaction was terminated after 20 min by the addition of acetonitrile (5 ml). Samples were vortexed on a SP multi-tube vortexer (Baxter, McGraw Park, IL, USA) for 10 min, followed by centrifugation at 2000 ***g*** for 10 min at room temperature. The organic layer was removed and evaporated under a gentle stream of nitrogen using a Dri-Block sample concentrator (Techne, Princeton, NJ, USA) at 50°C. Samples were reconstituted in 200 μl in 60% acetonitrile/40% 10 mM ammonium phosphate buffer, pH 4.0.

Paclitaxel and $6^{\alpha}$-hydroxypaclitaxel were measured by a previously published method (Jamis-Dow et al, 1995). Testosterone metabolism was monitored by high-performance liquid chromatography (HPLC), with UV detection at 254 nm (Lillibridge et al, 1998).

Pharmacokinetic parameters were estimated by non-compartmental analysis. Area under the concentration-time curve (AUC) for plasma was calculated using a combination of trapezoid and log-trapezoid methods. Half-life was calculated from the terminal, log-linear portion of the plasma concentration–time curve.

### RESULTS

### In vitro combination cytotoxicity studies

From the single-agent in vitro studies performed, the following $IC_{50}$s were calculated (mean (s.d.) $n = 3$): 3-h paclitaxel exposure; HNX14C 0.031 (±0.006) μM, HNX22B 0.038 (±0.005) μM; 120-h nolatrexed exposure; HNX14C 1.12 (±0.06) μM, HNX22B 0.66 (±0.06) μM.

For schedule 1 the combination of paclitaxel and nolatrexed in vitro demonstrated antagonistic effects for both cell lines, as demonstrated in Figures 1 and 2. For HNX14C the pattern on schedule 2 was different. When cell growth was measured on day 6 there was primarily antagonism, with some synergistic effects, but only at high levels of growth inhibition (Figure 1A). However, when growth was measured on day 8, there was marked synergism throughout the range of Fa (Figure 1A). For the HNX22B cell line treated with schedule 2, some synergism was again observed, but only at values of Fa greater than 0.5 (Figure 2A). Indeed the combination appeared to behave in an antagonistic manner below this Fa value. With schedule 3 and for Fa values of less than 0.75 in the HNX22B cell line, antagonism was observed for the HNX22B cell line regardless of the day on which cell growth was determined (Figure 2B). Similarly, for schedule 3 in the HNX14C cell line, the appearance of the CI/Fa graphs for growth inhibition on days 6, 8 and 10 are almost identical and reveal antagonism up

*© 2000 Cancer Research Campaign*

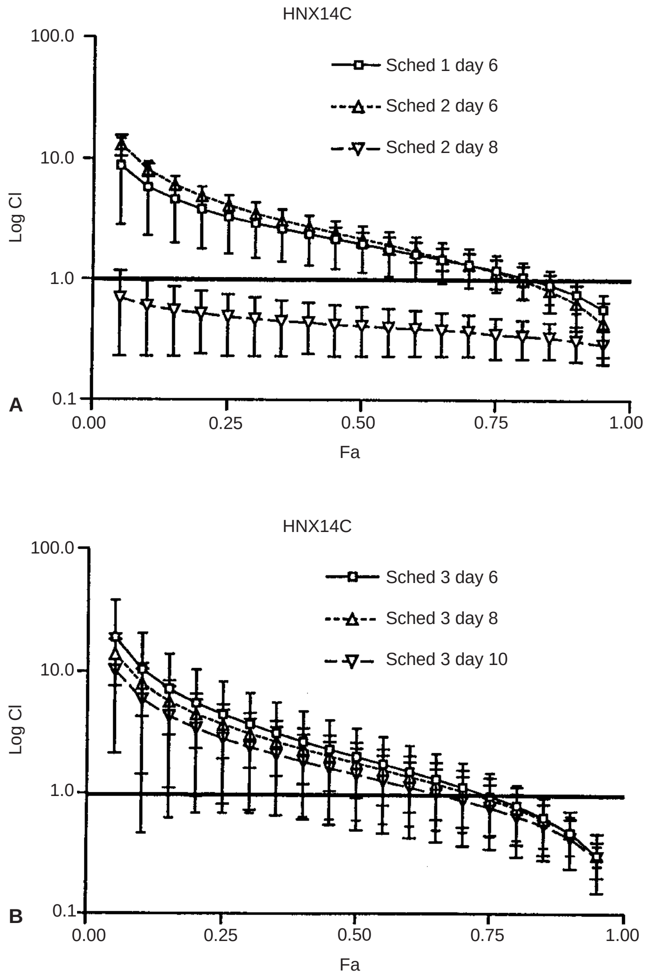

**Figure 1** In vitro growth inhibition studies with nolatrexed and paclitaxel combinations for the HNX14C cell line. Graphs of log combination index (CI) versus fraction of cells affected (Fa). Schedules 1 and 2 in (**A**) and schedule 3 in (**B**). The data points represent the mean value and standard deviation for a minimum of three experiments. A CI value = 1 implies an additive effect of the drug combination at that particular Fa. A value > 1 implies antagonism and < 1 synergism

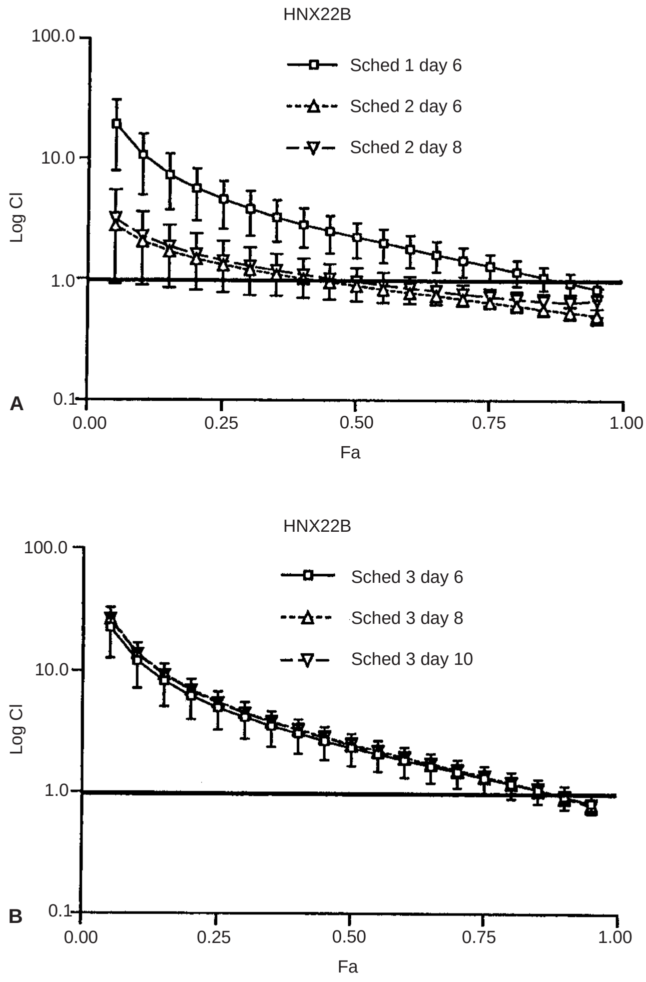

**Figure 2** In vitro growth inhibition studies with nolatrexed and paclitaxel combinations for the HNX22B cell line. Graphs of log combination index (CI) versus fraction of cells affected (Fa). Schedules 1 and 2 in (**A**) and schedule 3 in (**B**). See Figure 1 for interpretation to a Fa value of approximately 0.75 after which synergistic effects predominate (Figure 1B). In conclusion, the in vitro data showed some evidence of synergy on schedule 2, particularly in the HNX14C cells. However, the effect was not sufficiently consistent to preclude clinical assessment of all three schedules.

### Clinical study

### *Pharmacokinetics*

Full pharmacokinetic sampling was performed on the first course for each of nine patients. The pharmacokinetic parameters of interest were peak plasma concentration, AUC, clearance and half-life of both nolatrexed and paclitaxel. In addition, the peak concentration and AUC of the two main paclitaxel metabolites was measured, i.e. those caused by 6-hydroxylation and 3′phenyl-*p*-hydroxylation of the parent compound. Plasma concentration-time curves of nolatrexed and paclitaxel for a typical patient on each schedule are represented in Figure 3. The paclitaxel pharmacokinetic data are shown in Table 2 with the corresponding data for nolatrexed in Table 3.

Whilst nolatrexed pharmacokinetics were unaffected by schedule, a schedule-dependent interaction of nolatrexed with paclitaxel was observed. Typical pharmacokinetic parameters for paclitaxel when administered as a single agent (Keung et al, 1993; Gianni et al, 1995) are shown in Table 2. Patients on schedule 2 had a lower clearance of paclitaxel (165–238 ml min$^{-1}$ m$^{-2}$) when compared to patients on the other 2 schedules (322–520 ml min$^{-1}$ m$^{-2}$), with the exception of patient 8. The paclitaxel clearance on schedule 2 was comparable to that observed with a single-agent dose of 175 mg m$^{-2}$ and, as a result of the lower clearance on schedule 2, peak plasma concentrations (1.66–1.93 μM versus 0.86–1.32 μM) and AUCs (392–565 μM min$^{-1}$ versus 180–291 μM min$^{-1}$) were higher. The AUC achieved with 80 mg m$^{-2}$ paclitaxel on schedule 2 approaches that with a single-agent dose of 135 mg m$^{-2}$. The exception to the above generalizations was patient 8 who, although treated on schedule 3, had pharmacokinetic parameters similar to patients on schedule 2. This patient subsequently received single-agent paclitaxel (135 mg m$^{-2}$ as a 3-h infusion) and, in the absence of nolatrexed, impaired elimination of paclitaxel was still observed (CI = 127 ml min$^{-1}$ m$^{-2}$).

 © 2000 Cancer Research Campaign

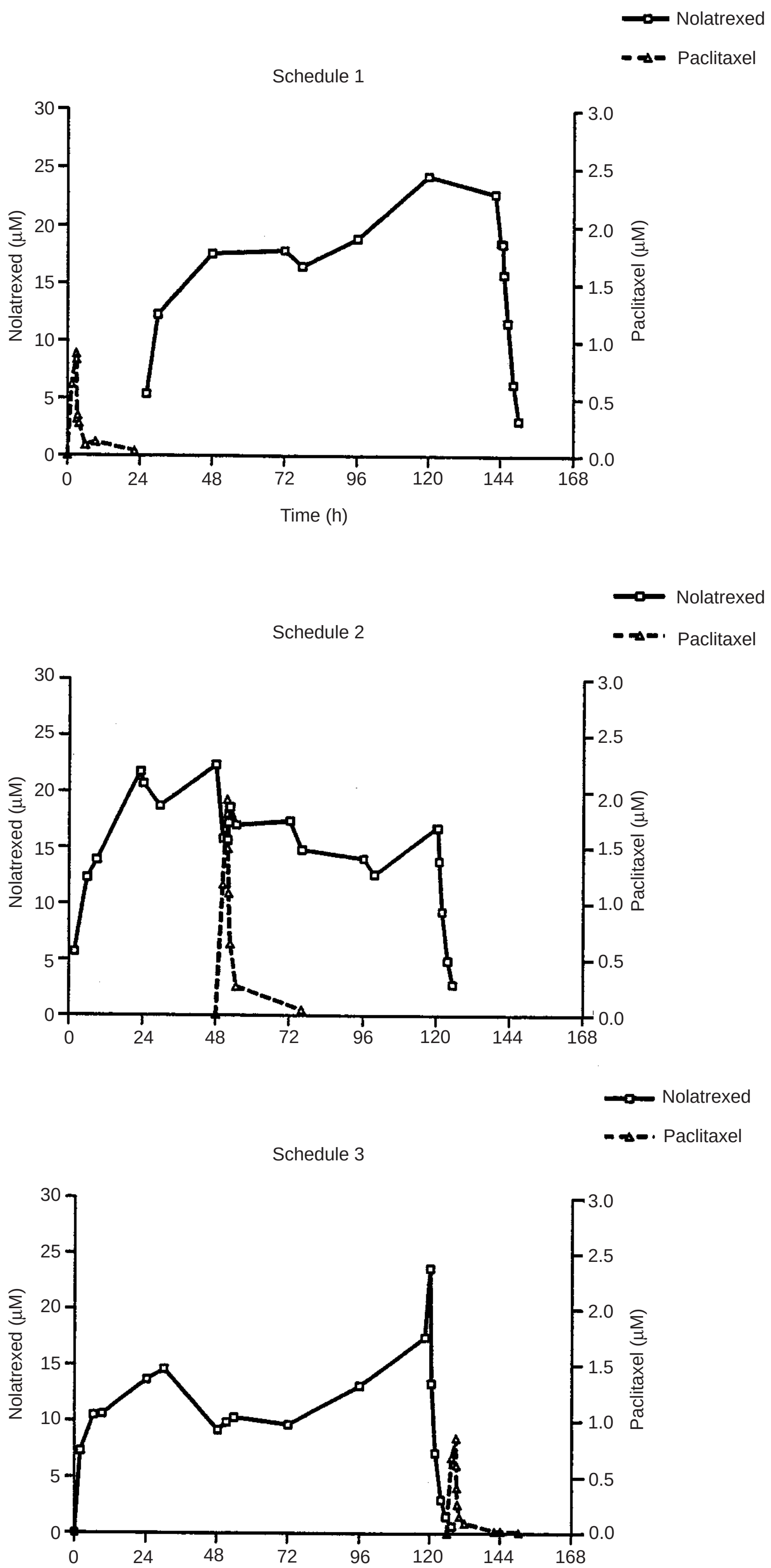

**Figure 3** Plasma drug concentration versus time profiles for both paclitaxel and nolatrexed in a typical patient for each schedule of drug administration. Upper panel schedule 1, middle panel schedule 2, lower panel schedule 3

Paclitaxel metabolite peaks were identified by retention time (Huizing et al, 1993) and peak concentrations and AUCs were calculated. Paclitaxel metabolite AUCs were lowest in the patients treated on schedule 2. Patient 8, on schedule 3, had high levels of both metabolites, both when paclitaxel was administered alone and in combination with nolatrexed, suggesting a deficiency in secondary metabolism or biliary elimination of paclitaxel.

**Table 2** The pharmacokinetics of paclitaxel and its metabolites given at a dose of 80 mg m $^{-2}$ in combination with nolatrexed

| | | Paclitaxel | | | | | 6-OH metabolite | | 3′ phenyl-hydroxy metabolite | |
|---|---|---|---|---|---|---|---|---|---|---|
| Patient no | Schedule | Dose (mg) | Peak conc. (μM) | AUC (μM min$^{-1}$) | Clearance (ml min$^{-1}$ m$^{-2}$) | Half-life (min) | Peak conc. (μM) | AUC (μM min$^{-1}$) | Peak conc. (μM) | AUC (μM min$^{-1}$) |
| 3 | 1 | 126 | 1.00 | 227 | 411 | 421 | 0.26 | 120 | 0.37 | 162 |
| 6 | 1 | 146 | 0.89 | 249 | 376 | 442 | 0.05 | 23 | 0.06 | 18 |
| 9 | 1 | 131 | 1.32 | 291 | 322 | 507 | 0.06 | 19 | ND | ND |
| 5 | 2 | 114 | 1.93 | 565 | 165 | 413 | 0.02 | 11 | ND | ND |
| 7 | 2 | 146 | 1.66 | 392 | 238 | 381 | 0.02 | 8 | ND | ND |
| 10 | 2 | 148 | 1.76 | 547 | 171 | 416 | 0.01 | 3 | ND | ND |
| 2 | 3 | 124 | 0.86 | 180 | 520 | 365 | ND | ND | ND | ND |
| 4 | 3 | 143 | 0.91 | 187 | 500 | 505 | 0.05 | 8 | 0.02 | 11 |
| 8 | 3 | 121 | 2.97 | 606 | 155 | 381 | 0.42 | 193 | 0.16 | 102 |
| *8 | N/A | 205 | 5.06 | 1239 | 127 | 360 | 0.79 | 217 | 0.4 | 172 |
| Gianni, 1995 | 135 mg m$^{-2}$ | 3.30 | 654 | 247 | | | | | | |
| Keung, 1993 | 135 mg m$^{-2}$ | 2.50 | 564 | 295 | | | | | | |

*These results are for patient 8 treated with single-agent paclitaxel (dose 135 mg m$^{-2}$). See text for explanation. AUC = area under plasma concentration-time curve. ND = metabolite peak not detected.

**Table 3** The pharmacokinetics of nolatrexed given at a dose of 500 mg m$^{-2}$ day$^{-1}$ in combination with paclitaxel

| Patient no. | Schedule | Total dose (mg) | Peak conc. (μg ml$^{-1}$) | AUC (mg ml$^{-1}$ min$^{-1}$) | Clearance (ml min$^{-1}$ m$^{-2}$) | Half-life (min) |
|---|---|---|---|---|---|---|
| 3 | 1 | 3940 | 6.2 | 18 | 137 | 66 |
| 6 | 1 | 4575 | 6.9 | 39 | 64 | 113 |
| 9 | 1 | 4100 | 6.7 | 36 | 70 | 128 |
| 5 | 2 | 3575 | 6.4 | 34 | 73 | 118 |
| 7 | 2 | 4575 | 6.6 | 23 | 108 | 95 |
| 10 | 2 | 4625 | 5.0 | 28 | 91 | 154 |
| 2 | 3 | 3875 | 6.7 | 26 | 99 | 97 |
| 4 | 3 | 4225 | 8.5 | 38 | 62 | 132 |
| 8 | 3 | 3800 | 5.6 | 32 | 79 | 114 |

AUC = area under plasma concentration/time curve.

### *In vitro metabolism studies*

Nolatrexed inhibited the metabolism of paclitaxel to its 6-hydroxy-metabolite with a $K_i$ of 0.64 μM. In addition, nolatrexed inhibited the metabolism of the model CYP3A4 substrate testosterone, with a $K_i$ of 2.7 μM.

### *Toxicity*

The toxicities experienced by the patients on all 22 courses of therapy are summarized in Table 4. Only two courses were complicated by myelosuppression. Patient 8 had grade 4 neutropenia, grade 3 leucopenia and grade 2 thrombocytopenia on her only course of treatment. She had metastatic breast carcinoma and had previously been treated with six cycles of epirubicin and cyclophosphamide. As shown in Table 3, it is highly likely that the toxicity can be explained by the pharmacokinetics of paclitaxel in this patient. Patient 10 suffered grade 3 neutropenia with his only course of treatment, although this did not require medical intervention. He had metastatic caecal carcinoma and had previously received 5-fluorouracil and folinic acid chemotherapy. Again, this toxicity appeared to be related to the pharmacokinetics of paclitaxel as this patient was treated on schedule 2.

From the viewpoint of gastrointestinal side-effects, nine of 22 courses were complicated by toxicity of grade 3 or greater. Patient

**Table 4** Toxicity of the combination of paclitaxel and nolatrexed.

| | Grade | | | |
|---|---|---|---|---|
| Toxicity | 1 | 2 | 3 | 4 |
| WBC | | | 1 | |
| Platelets | | 1 | | |
| Neutrophils | | | 1 | 1 |
| Nausea | 8 | 3 | 3 | |
| Vomiting | 1 | 1 | | 2 |
| Diarrhoea | | 5 | 1 | |
| Mucositis | 5 | 4 | 3 | |
| Neurosensory | 6 | | | |
| Rash | 2 | 4 | | |
| Fatigue | 1 | 15 | | |
| Alopecia | | 2 | | |
| Arthralgia | 4 | 2 | | |
| *Others | 6 | 7 | 3 | |

Data from all three schedules have been pooled. (Figures represent the number of patient courses in which a particular grade of toxicity was experienced.) *Other toxicities included facial flushing, altered taste, dyspepsia, pruritus and myalgia.

 *© 2000 Cancer Research Campaign*

2 experienced grade 3 nausea and grade 4 vomiting and was taken off study because of this. This patient required intravenous anti-emetic therapy, but had experienced similar problems prior to receiving any treatment. There was no obvious association between the gastrointestinal toxicity and schedule of treatment.

A grade 2 rash was experienced in four patient courses and grade 2 fatigue in the majority of courses. Interestingly, neither of the two chemotherapy-naive patients experienced any toxicity greater than grade 2. Between them, they received six of the 22 courses of treatment.

### *Efficacy*

Of the nine patients entered on the study, only six were evaluable for response, receiving a total of 19 courses of treatment. Three of these patients had stable disease after a minimum of four courses. The other three patients progressed whilst on treatment. No responses were observed on the study.

### DISCUSSION

The aim of this study was to determine the optimum schedule and pharmacology of the combination of paclitaxel and nolatrexed in the treatment of solid tumours, particularly head and neck cancer. As well as potential interactions in terms of anti-tumour effect and host toxicity, the potential for pharmacokinetic interactions must also be considered.

The metabolism of paclitaxel has been widely studied in humans. Hepatic metabolism by the cytochrome P450 (CYP) system and biliary excretion play an important role in the removal of paclitaxel from the plasma (Kearns, 1997). The two major metabolites observed in humans are 6-hydroxy paclitaxel, whose formation is mediated by CYP2C8 (Cresteil et al, 1994) and 3′-phenyl-*p*-hydroxy paclitaxel mediated by CYP3A4 (Cresteil et al, 1994; Sonnichsen et al, 1995). Cytochrome P450 inducers such as phenytoin and phenobarbitone would be expected to increase the metabolism of paclitaxel and this has been shown in studies using human liver preparations (Harris et al, 1994) and in patients (Kuhn et al, 1997). Cytochrome P450 inhibitors, such as ketoconazole and fluconazole, have been shown to inhibit the metabolism of paclitaxel in in vitro studies (Jamis-Dow et al, 1997). However, no metabolic interactions have been reported with the drugs commonly used in the paclitaxel pre-medication regime, notably dexamethasone, cimetidine and chlorpheniramine (Jamis-Dow et al, 1995; Schlichenmyer et al, 1995). Paclitaxel clearance is reduced when administered with multi-drug resistance reversal agents (Berg et al, 1995).

In the current study, patients on schedule 2 who received paclitaxel concurrently with nolatrexed had a lower clearance of paclitaxel with higher peak paclitaxel plasma concentrations and AUCs. In vitro studies demonstrated that nolatrexed was a potent inhibitor of CYP2C8-mediated metabolism of paclitaxel ($K_i$ 0.64 μM) and of CYP3A4 ($K_i$ 2.70 μM) which catalyse the two major routes of paclitaxel metabolism. Thus, nolatrexed apparently reduces the elimination of paclitaxel by inhibiting CYP450-mediated metabolism. There was no schedule-dependent effect on nolatrexed pharmacokinetic profiles when combined with paclitaxel.

On schedules 1 and 3, paclitaxel pharmacokinetics were in accord with previous data. The greater clearance of paclitaxel observed, compared to literature values at higher doses, is consistent with the reported non-linearity in paclitaxel pharmacokinetics. The exception to this was patient 8, who was suffering from breast carcinoma that had metastasized to the liver and bones. Liver function tests showed a bilirubin of 13 (normal range up to 18), alkaline phosphatase of 638 (normal up to 120), AST of 87 (0–40) and ALT of 116 (0–40). Hepatic metabolism and biliary excretion are the main elimination pathways of paclitaxel in humans, and paclitaxel metabolism is known to be impaired in patients with decreased hepatic function (Wilson et al, 1994; Panday et al, 1997; Chao et al, 1998; Venook et al, 1998). There is growing evidence that dose reductions of paclitaxel should be routinely performed in patients with altered hepatic function in order to avoid excessive toxicity (Panday et al, 1997; Venook et al, 1998). After administration of single-agent paclitaxel (135 mg $m^{-2}$ given as a 3-h infusion) to patient 8, impaired elimination was again observed, supporting the lack of an interaction between nolatrexed and paclitaxel and impaired hepatic function as the most likely explanation for the reduced paclitaxel clearance.

In previous studies, schedule-dependent effects have been observed in clinical trials involving paclitaxel. When cisplatin was administered before paclitaxel, more severe neutropenia was observed than if the reverse schedule was used. The greater toxicity appeared to be due to reduced clearance of paclitaxel when it was given after cisplatin, an effect that was attributed to the modulatory effect of cisplatin on cytochrome P450 enzymes (Rowinsky et al, 1991). Conversely, myelosuppression has been shown to be more severe when paclitaxel precedes cyclophosphamide administered in combination (Kennedy et al, 1998), and it has been reported that paclitaxel reduces doxorubicin elimination (Gianni et al, 1997). Previous work has stressed the importance of the time spent above certain critical plasma paclitaxel concentrations, particularly in relation to predicting myelosuppression. The threshold concentration has been reported to be either 0.1 (Sonnichsen and Relling, 1994) or 0.05 μM (Vigano et al, 1995). Non-haematological (e.g. neuropathy) toxicities also relate more closely to paclitaxel AUC and time spent above critical plasma concentrations than with the administered dose. In the current study, times above 0.1 μM were longer for schedule 2 (10–18 h) compared to schedules 1 and 3 (5–8 h), and for times above 0.05 μM a similar difference was seen (16–27 h compared to 8–18 h).

The in vitro combination studies demonstrated a potential advantage for schedule 2, where a synergistic effect was observed in at least one cell line. Conversely, for schedules 1 and 3, an antagonistic profile dominated. Previous in vitro studies have also shown schedule-dependent effects for paclitaxel in combination with other chemotherapeutic agents. For example, in human gastric and ovarian carcinoma cell lines, the combination of cisplatin and paclitaxel was found to be either additive or even synergistic when paclitaxel administration preceded that of cisplatin by a 24-h period (Vanhoefer et al, 1995). Conversely, when the drugs were co-administered or when cisplatin was given before paclitaxel, strong antagonism was observed. Schedule-dependency has also been examined using the combination of paclitaxel and SN-38 (the active metabolite of irinotecan) and four human cancer cell lines where additive effects were observed regardless of the sequence of treatment (Kano et al, 1998). However, simultaneous administration of the two drugs produced antagonistic effects in three of the four cell lines. In vitro studies with paclitaxel and 5-FU demonstrated antagonism when 5-FU was administered prior to paclitaxel but more than additive cytotoxicity with the reverse schedule (Geoffroy et al, 1994).

*© 2000 Cancer Research Campaign*

Clinically, three different schedules of administration of nolatrexed and paclitaxel were studied, and all were well-tolerated. Myelosuppression was rare with the major side-effect being gastrointestinal toxicity. Fatigue was also common, affecting the majority of patient courses. No responses were seen, although three patients had stable disease.

In summary, the metabolism and hence clearance of paclitaxel is impaired when the drug is co-administered with nolatrexed. Further investigation of schedules and dosing was discontinued because the combination of in vitro and in vivo data indicated that the schedule most likely to be active produced a pharmacokinetic interaction. Further exploration of this drug combination would require careful dose optimization, especially given the non-linear pharmacokinetics of both drugs.

### ACKNOWLEDGEMENTS

The authors would like to acknowledge the assistance of the Research Sisters, Fiona Chapman, Dorothy Simmons and Madeline Proctor and data manager Kevin Fishwick at Newcastle General Hospital. This work was made possible by financial support from the Cancer Research Campaign and Agouron Pharmaceuticals.

### REFERENCES

Aisner J and Cortes-Funes H (1997) Paclitaxel in head and neck and other cancers: future prospects. *Semin Oncol* **24**: 113–115

Belani CP, Agarwala S, Johnson J, Cohn A, Bernstein J, Langer C, Jones V, White C, Loh K, Whiter D, Chew T, Johnston A and Clendeninn N (1997*a*) A phase II trial with Thymitaq™ (AG337) in patients with squamous cell carcinoma of the head and neck. *Proc ASCO* **16**: 387a

Belani CP, Lembersky B, Ramanathan R, Cohn A, Loh K, Miller W, Green M, Chew T, Johnston A and Clendeninn N (1997*b*) A phase II trial of Thymitaq™ (AG337) in patients with adenocarcinoma of the colon. *Proc ASCO* **16**: 272a

Berg SL, Tolcher A, O'Shaughnessy JA, Denicoll AM, Noone M, Ognibene FP, Cowan KH and Balis FM (1995) Effect of R-verapamil on the pharmacokinetics of paclitaxel in women with breast cancer. *J Clin Oncol* **13**: 2039–2042

Chao Y, Chan WK, Birkhofer MJ, Hu OJ, Wang SS, Huang YS, Liu M, Whang-Peng J, Chi KH, Lui WY and Lee SD (1998) Phase II and pharmacokinetic study of paclitaxel therapy for unresectable hepatocellular carcinoma patients. *Br J Cancer* **78**: 34–39

Chou TC and Talalay P (1984) Quantitative analysis of dose–effect relationships: the combined effects of multiple drugs or enzyme inhibitors. *Adv Enzyme Reg* **22**: 27–55

Cresteil T, Monsarrat B, Alvinerie P, Treluyer JM, Vieira I and Wright M (1994) Taxol metabolism by human liver microsomes: identification of cytochrome P450 isozymes involved in its biotransformation. *Cancer Res* **54**: 386–393

D'Andrea GM and Seidman AD (1997) Docetaxel and paclitaxel in breast cancer therapy: present status and future prospects. *Semin Oncol* **24**: S1327–1344

Geoffroy F, Patel M, Ren Q-F and Grem J (1994) Interaction of fluorouracil and paclitaxel in MCF-7 human breast cancer. *Proc AACR* **35**: 330 (abstract 1962)

Gianni L, Kearns C, Giani A, Capri G, Vigano L, Locatelli A, Bonadonna G and Egorin M (1995) Non-linear pharmacokinetic and metabolism of paclitaxel and its pharmacokinetic/pharmacodynamic relationships in humans. *J Clin Oncol* **13**: 180–190

Gianni L, Vigano L, Locatelli A, Capri G, Giani A, Tarenzi E and Bonadonna G (1997) Human pharmacokinetic characterization and in vitro study of the interaction between doxorubicin and paclitaxel in patients with breast cancer. *J Clin Oncol* **15**: 1906–1915

Goldspiel BR (1997) Clinical overview of the taxanes. *Pharmacotherapy* **17**: 110–125

Harris JW, Rahman A, Kim B-R, Guengerich FP and Collins JM (1994) Metabolism of Taxol by human hepatic microsomes and liver slices: Participation of cytochrome P450 3A4 and an unknown P450 enzyme. *Cancer Res* **54**: 4026–4035

Huizing MT, Keung ACF, Rosing H, van der Kuij V, ten Bokkel-Huinink WW, Mandjes IM, Dubbelman ARC, Pinedo HM and Beijnen JH (1993) Pharmacokinetics of paclitaxel and metabolism in a randomised comparative study in platinum-pretreated ovarian cancer patients. *J Clin Oncol* **11**: 2127–2135

Jamis-Dow CA, Klecker RW, Katki AG and Collins JM (1995) Metabolism of taxol by human and rat liver in vitro: A screen for drug interactions and interspecies differences. *Cancer Chemother Pharmacol* **36**: 107–114

Jamis-Dow CA, Pearl ML, Watkins PB, Blake DS, Klecker RW and Collins JM (1997) Predicting drug interactions in vivo from experiments in vitro – Human studies with paclitaxel and ketoconazole. *Am J Clin Oncol – Cancer Clin Trials* **20**: 592–599

Kano Y, Akutsu M, Tsunoda S, Mori K, Suzuki K and Adachi KI (1998) In vitro schedule-dependent interaction between paclitaxel and SN-38 (the active metabolite of irinotecan) in human carcinoma cell lines. *Cancer Chemother Pharmacol* **42**: 91–98

Kearns CM (1997) Pharmacokinetics of the taxanes. *Pharmacotherapy* **17**: 105S–109S

Kennedy MJ, Zahurak ML, Donehower RC, Noe D, Grochow LB, Sartorius S, Chen T-L, Bowling K, Duerr M and Rowinsky EK (1998) Sequence-dependent hematological toxicity associated with the 3-hour paclitaxel/cyclophosphamide doublet. *Clin Cancer Res* **4**: 349–356

Keung ACF, Kaul S, Pinedo HM, ten Bokkel Huinink WW and Beijnen JH (1993) Pharmacokinetics of taxol given by 3-h or 24-h infusion to patients with ovarian carcinoma. *Proc ASCO* **12**: 130 (abstract 321)

Kuhn J, Rizzo J, Chang S, Schold C, Spence A, Berger M, Robins H, Mehta M, Bozik M, Fulton D, Rector D and Prados M (1997) Effects of anticonvulsants on the pharmacokinetics and metabolic profile of paclitaxel. *Proc ASCO* **16**: 224 (abstract 787)

Lillibridge JH, Liang BH, Kerr BM, Webber S, Quart B, Shetty BV and Lee CA (1998) Characterization of the selectivity and mechanism of human cytochrome P450 inhibition by the human immunodeficiency virus-protease inhibitor nelfinavir mesylate. *Drug Metab Disp* **26**: 609–161

Loh K, Stuart K, Cohn A, White C, Hines J, Miller W, Green MR, Chew T, Johnston A and Clendeninn N (1997) A Phase II trial of Thymitaq™ (AG337) in patients with adenocarcinoma of the pancreas. *Proc ASCO* **16**: 265a

Panday VR, Huizing MT, Willemse PH, De Graeff A, ten Bokkol Huinink WW, Vermorken JB and Beijnen JH (1997) Hepatic metabolism of paclitaxel and its impact in patients with altered hepatic function. *Semin Oncol* **24**: S11–S38

Rafi I, Taylor GA, Calvete JA, Boddy AV, Balmanno K, Bailey N, Lind M, Calvert AH, Webber S, Jackson RC, Johnston AJ, Clendeninn N and Newell DR (1995) Clinical pharmacokinetic and pharmacodynamic studies with the non-classical antifolate thymidylate synthase inhibitor 3,4-dihydro-2-amino-6-methyl-4-oxo-5-(4-pyridylthio)-quinazolone dihydrochloride (AG337) given by 24-hour continuous intravenous infusion. *Clin Cancer Res* **1**: 1275–1284

Rafi I, Boddy AV, Calvete JA, Taylor GA, Newell DR, Bailey NP, Lind MJ, Green M, Hines J, Johnstone A, Clendeninn N and Calvert AH (1998) Preclinical and phase I clinical studies with the nonclassical antifolate thymidylate synthase inhibitor nolatrexed dihydrochloride given by prolonged administration in patients with solid tumors. *J Clin Oncol* **16**: 1131–1141

Rowinsky EK (1997) Paclitaxel: dosing and schedule issues. *Oncology* **11**: 7–19

Rowinsky EK, Gilbert MR, McGuire WP, Noe DA, Grochow LB, Forastiere AA, Ettinger DS, Lubejko BG, Clark B, Sartorius SE, Cornblath DR, Hendricks CB and Donehower RC (1991) Sequences of taxol and cisplatin: A phase I and pharmacologic study. *J Clin Oncol* **9**: 1692–1703

Schlichenmyer WJ, Donehower RC, Chen TL, Bowling MK, McQuire WP and Rowinsky, E K (1995) Pre-treatment H2 receptor antagonists that differ in P450 modulation activity: comparative effects on paclitaxel clearance rates and neutropenia. *Cancer Chemother Pharmacol* **36**: 227–232

Siddiqui N, Boddy AV, Thomas HD, Bailey NP, Robson L, Lind MJ and Calvert AH (1997) A clinical and pharmacokinetic study of the combination of carboplatin and paclitaxel for epithelial ovarian cancer. *Br J Cancer* **75**: 287–294

Skehan P, Storeng R, Scudiero D, Monks A, McMahon J, Vistica D, Warren JY, Bokesch H, Kenney S and Boyd MR (1990) New colorimetric cytotoxicity assay for anticancer-drug screening. *J Natl Cancer Inst* **82**: 1107–1112

Sonnichsen D and Relling M (1994) Clinical pharmacokinetics of paclitaxel. *Clin Pharmacokin* **27**: 256–269

Sonnichsen DS, Liu Q, Schuetz EG, Schuetz JD, Pappo A and Relling MV (1995) Variability in human cytochrome P450 paclitaxel metabolism. *J Pharmacol Exp Ther* **275**: 566–575

Stuart KE, Hajdenberg J, Cohn A, Loh KK, Miller W, White C and Clendeninn NJ (1996) A Phase II trial of Thymitaq™ (AG337) in patients with hepatocellular carcinoma. Proc *ASCO* **15**: 202

Vanhoefer U, Harstrick A, Wilke H, Schleucher N, Walles H, Schroder J and Seeber S (1995) Schedule-dependent antagonism of paclitaxel and cisplatin in human gastric and ovarian carcinoma cell lines in vitro. *Eur J Cancer* **31A**: 92–97

 © 2000 Cancer Research Campaign

Venook AP, Egorin MJ, Rosner GL, Brown TD, Jahan TM, Batist G, Hohl R, Budman D, Ratain MJ, Kearns CM and Schilsky RL (1998) Phase I and pharmacokinetics trial of paclitaxel in patients with hepatic dysfunction. *J Clin Oncol* **16**: 1811–1819

Vigano L, Lacatelli A, Bonadonna G and Egorin MJ (1995) Nonlinear pharmacokinetics and metabolism of paclitaxel and its pharmacokinetic/pharmacodynamic relationships in humans. *J Clin Oncol* **13**: 180–190

Vokes EE, Haraf DJ, Stenson K, Stupp R, Malone D, Levin J and Weichelsbaum RR (1995) The role of paclitaxel in the treatment of head and neck cancer. *Semin Oncol* **22 S12**: 8–12

Webber SE, Bleckman TM, Attard J, Deal JG, Kathardekar V, Welsh KM, Webber S, Janson CA, Matthews DA, Smith WW, Freer ST, Jordan SR, Bacquet RJ, Howland, EF, Booth CLJ, Ward RW, Hermann SM, White J, Morse CA, Hilliard JA and Bartlett CA (1993) Design of thymidylate synthase inhibitors using protein crystal-structures – the synthesis and biological evaluation of a novel class of 5-substituted quinazolinones. *J Med Chem* **36**: 733–746

Wilson WH, Berg SL, Bryant G, Wittes RE, Bates S, Fojo A, Steinberg SM, Goldspiel BR, Herdt J and O'Shaughnessy J (1994) Paclitaxel in doxorubicin-refractory or mitoxantrone-refractory breast cancer: a phase I/II trial of 96-hour infusion. *J Clin Oncol* **12**: 1621–1629

*© 2000 Cancer Research Campaign*
